# Modeling Competitive Mixtures With the Lotka-Volterra Framework for More Complex Fitness Assessment Between Strains

**DOI:** 10.3389/fmicb.2020.572487

**Published:** 2020-09-22

**Authors:** Afonso Dimas Martins, Erida Gjini

**Affiliations:** ^1^Mathematical Modeling of Biological Processes Laboratory, Instituto Gulbenkian de Ciência, Oeiras, Portugal; ^2^Departamento de Estatística e Investigacão Operacional, Faculdade de Ciências, Universidade de Lisbon, Lisbon, Portugal

**Keywords:** microbial interactions, influenza, competitive mixture, relative transmission fitness, bottleneck size, multiple strains, frequency dependence, coinfection

## Abstract

With increasing resolution of microbial diversity at the genomic level, experimental and modeling frameworks that translate such diversity into phenotypes are highly needed. This is particularly important when comparing drug-resistant with drug-sensitive pathogen strains, when anticipating epidemiological implications of microbial diversity, and when designing control measures. Classical approaches quantify differences between microbial strains using the exponential growth model, and typically report a selection coefficient for the relative fitness differential between two strains. The apparent simplicity of such approaches comes with the costs of limiting the range of biological scenarios that can be captured, and biases strain fitness estimates to polarized extremes of competitive exclusion. Here, we propose a mathematical and statistical framework based on the Lotka-Volterra model, that can capture frequency-dependent competition between microbial strains within-host and upon transmission. As a *proof-of-concept*, the model is applied to a previously-published dataset from *in-vivo* competitive mixture experiments with influenza strains in ferrets (McCaw et al., 2011). We show that for the same data, our model predicts a scenario of coexistence between strains, and supports a higher bottleneck size in the range of 35–145 virions transmitted from donor to recipient host. Thanks to its simplicity and generality, such framework could be applied to other ecological scenarios of microbial competition, enabling a more complex and nuanced view of possible outcomes between two strains, beyond competitive exclusion.

## 1. Introduction

Microbial fitness estimation is an active area of research. Recent studies show expanded interest to connect *in-vitro* with *in-vivo* measurements (Mohapatra et al., 2012; Govorkova, 2013; Skurnik et al., 2013), within-host to between-host level processes (Hurt et al., 2010), and anticipating evolutionary trajectories of pathogens in response to host immunization or interventions, such as drugs and vaccines (Łuksza and Lässig, 2014; Neher et al., 2016). In viral research, given the increase in drug-resistance evolution (Oh and Hurt, 2014), vaccine escape, viral emergence, and host jumps, understanding viral fitness has become crucial (Wargo and Kurath, 2012; Domingo et al., 2019). Viral fitness estimation is typically based on statistical methods to compare replicative fitness of two viruses, in cultured cells, tissues, or in individual hosts. The field has expanded to include more sophisticated mathematical frameworks that consider particular life-history traits, and quantify replicative differences (Holder et al., 2011; Pinilla et al., 2012), as well as transmission fitness and subtle variation between strains (McCaw et al., 2011; Butler et al., 2014; Petrie et al., 2015). Estimating the transmission bottleneck size (Gutiérrez et al., 2012), which describes the size of the pathogen population transferred from the donor to the recipient host, has also received increasing attention (Leonard et al., 2017), because it may affect the rate of pathogen adaptation within host populations and their epidemiologic fitness. Ultimately, epidemiologic fitness of viruses (Domingo, 2010) and pathogens more generally results from the complex interplay with host immunity, heterogeneity, lifespan, and other environmental factors.

To address the challenge of bridging between within-host and between-host fitness of viruses, a novel experimental framework has been developed, known as the competitive mixture model (Hurt et al., 2010). The competitive mixtures model involves the infection of ferrets with a mixture of two different viruses [e.g., a wild-type (sensitive) and mutant (NAI-resistant) virus] and subsequent daily measurement of the relative proportions of those viruses to quantify whether one virus is replicating faster than the other. Typically, experiments involve the infection of “donor” ferrets with either a mixture (e.g., 80:20%, 50:50%, and 20:80%) or the pure virus, then, after 24 h, the infected donor is cohoused with a naive ferret (recipient 1). Once recipient 1 becomes infected, another round of transmission is enabled by cohousing it with another naive ferret (recipient 2). Daily nasal samples are collected and analyzed from ferrets to test whether the proportion of the two viruses within-host is changing over the course of infection, and upon transmission. Such data have been interpreted with mathematical models (Hurt et al., 2010; McCaw et al., 2011) to obtain a quantitative estimate of both the “within- host” (replication) and “between-host” (transmission) fitness of the mutant virus (compared to the wild type). A more recent modeling paper revisited this dataset (Petrie et al., 2015) with an explicit life-history modeling of viral dynamics within host and transmission. However, none of these frameworks accounts for the possibility of frequency-dependent fitness advantage of one strain over the other, in the chain of events from growth in donor to transmission to growth in recipient. In particular, by not including possible asymmetric interaction and density-dependent feedbacks between viral strains, such models can only describe scenarios of mutual exclusion.

**Figure 1:**
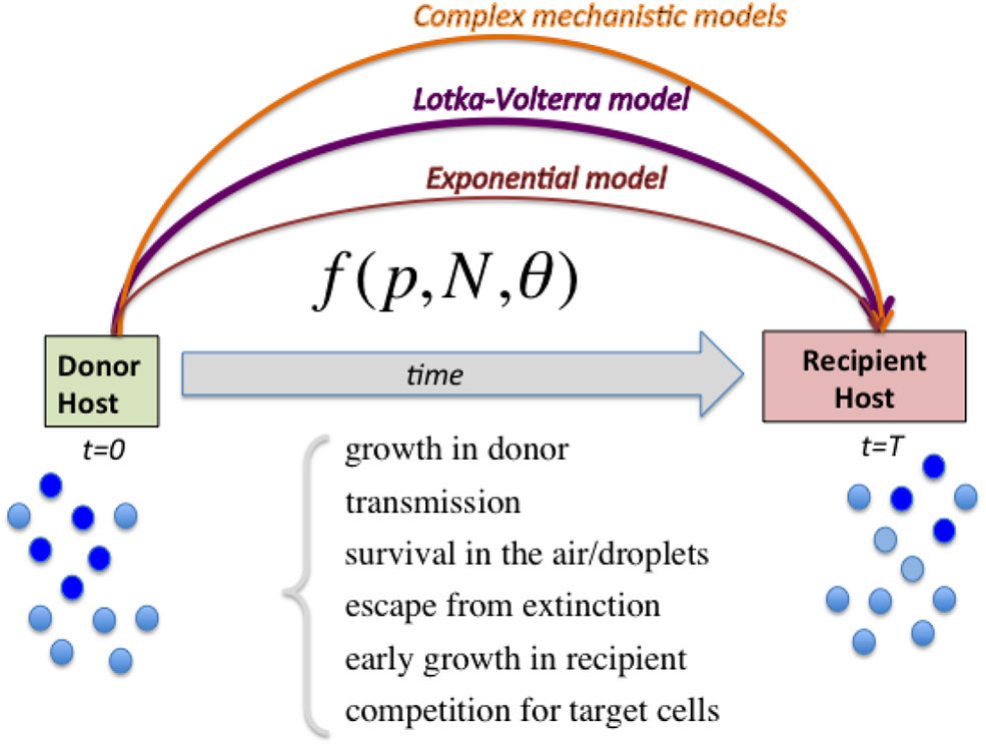
Diagram illustrating the range of models to capture relative fitness differences between strains at the growth-transmission interface. Linking the measured strain frequencies in the donor host (*p*) to the observed strain frequencies in recipient host after transmission, requires the specification of a model (here abstractly denoted by the function *f*) encapsulating multiple biological processes, and parameters encoding strain phenotypes θ as well as population size of viral load *N*. In order to harness the full information in competitive-mixture experiments (e.g., Hurt et al., 2010), alternatives to the purely exponential model for evaluating relative fitness between strains can be the Lotka-Volterra model formulation, as we advocate here, as well as more sophisticated models encoding explicit infection life-history traits. Complex models can capture more ecological scenarios, including bistability and coexistence, which lead to different predictions for within-host vs. between-host diversity in strain prevalences, and are likely to explain a wider range of epidemiological patterns.

In the present study we are motivated by such a limitation and propose to use a slightly more complex but yet simple and general enough model to account for more possible scenarios between two strains (Figure 1), namely the classical 2-species Lotka-Volterra model (Volterra, 1926). This model and its generalized form have a long history of use and application in the ecology of multi-species communities (MacArthur, 1970; May, 2001), and in particular also in microbial ecology and biology in recent years (see for example Stein et al., 2013; Shen et al., 2019). Given the generality of this framework, we believe it could prove useful also to understand interaction dynamics between viruses within-host, at least in the early time-scale before the immune response leading to infection clearance has been sufficiently activated (Tamura and Kurata, 2004). As a *proof-of-concept*, we apply this framework to the same dataset analyzed by McCaw et al. (2011) and estimate different parameters for fitness differences between the two influenza virus variants. Accounting for the possibility of stochastic transmission, with this model, we also estimate an average bottleneck size in the range of 45–135 virions, compatible with the larger estimates expected from the literature (Poon et al., 2016; Leonard et al., 2017).

By virtue of its simplicity, versatility and generality, we propose the Lotka-Volterra model to be used more widely in interpreting competitive-mixture experiments as the logical next-order extension, beyond the binary exponential model. We foresee applications of such quantitative framework for viral or bacterial strain transmission.

## 2. Materials and Methods

### 2.1. Classical Exponential Model

**Figure 2:**
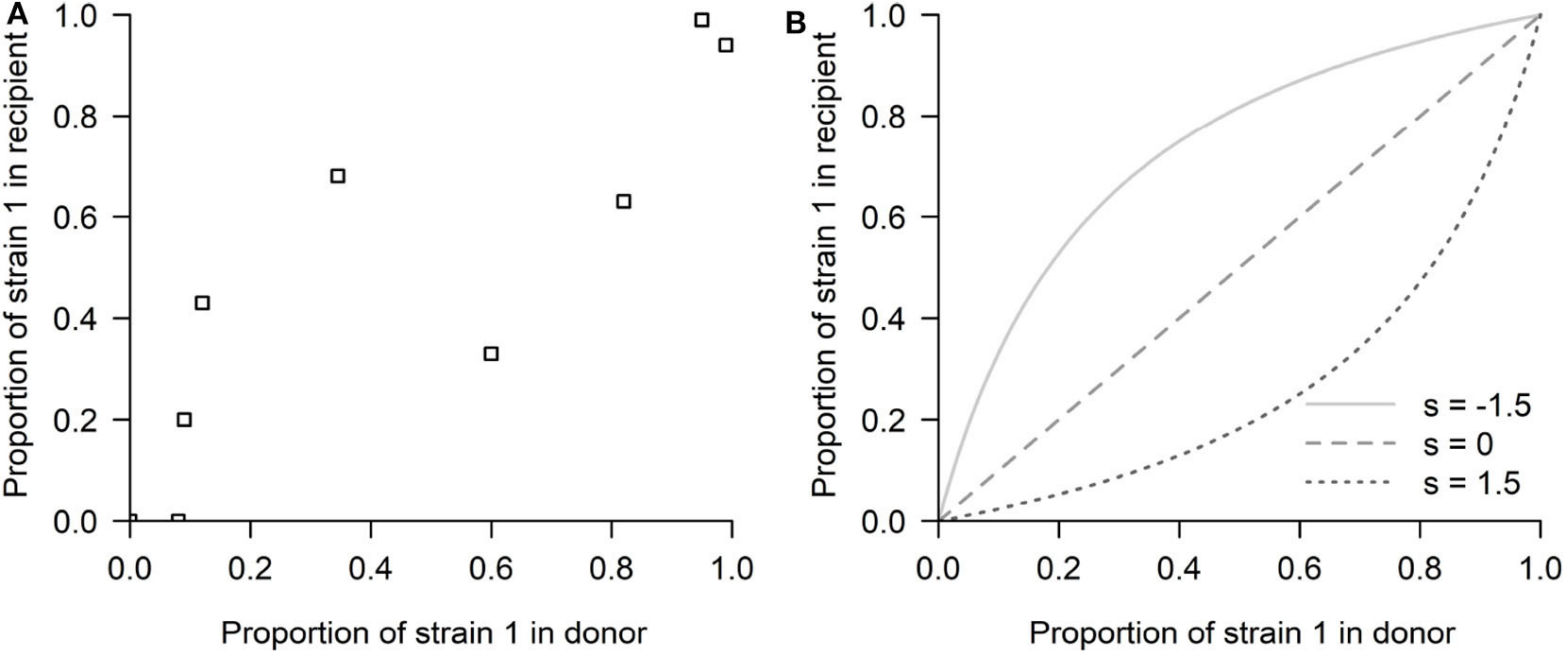
The competitive-mixtures data and the exponential model. **(A)** The donor-recipient data for competitive mixture transmission of influenza viruses analyzed by McCaw et al. (2011) (reproduced using the R software, after Figure 7 in Hurt et al., 2010). The abscissa shows the mutant proportion of the infecting ferret's viral load on the day preceding confirmed transmission. The ordinate shows the mutant proportion of the infected ferret's viral load within the first 24 h post-infection. Thus, the time passed between the two observations can be approximated to be 2 days. **(B)** The exponential model possibilities depending on the selection coefficient *s*: (*s* < 0) Mutant advantage, or (*s* > 0) WT advantage, thus expecting all the points above or below the diagonal, in a competitive exclusion scenario.

Our study was motivated by a series of experiments (Hurt et al., 2010), in which influenza strain transmission from donor to recipient was quantified in ferrets. The innovative nature of this experimental design was in quantifying the differences in transmission fitness between two strains using competitive mixtures. In particular, the study focused on an antiviral susceptible H1N1 strain and a resistant mutant strain (H274Y H1N1). Ferrets were inoculated with different proportions of these two strains and put in contact with other naive ferrets. In a later theoretical study, McCaw et al. (2011) modeled the relation between recipient ferret mutant proportion and donor ferret mutant proportion (see Figure 2A) with a simple model of mixture transmission, using the following expression:

(1) $$\begin{array}{l} P=\frac{p}{p+\left(1-p\right)e^{s}},\;-\infty <s<\infty \end{array}$$

where *P* is the mutant proportion in the recipient, *p* the mutant proportion in the donor and *s* a parameter of relative fitness advantage of the mutant strain relative to the wild-type strain, where *s* encompasses several layers of within-host and transmission fitness differences (probability to escape extinction, different growth rates, etc.) If *s* < 0, the mutant has a net fitness advantage relative to the wild-type, and the opposite if *s* > 0, the WT wins. This framework is a simple and useful approximation to compare the relative transmissibility fitness of two influenza strains. Indeed, with this framework, McCaw et al. (2011) were able to estimate a selection coefficient of *s* = −0.25, but for this particular data, with rather wide confidence intervals (−1.3509, 0.8329), including 0. Furthermore, when estimating the bottleneck size, the model structure inevitably biases the estimate to very small bottleneck size (*N*_*b*_ = 3.8), assigning the spread in the data to stochasticity, a finding that has been challenged as too low by other studies modeling influenza transmission in horses and pigs (Stack et al., 2013) and humans (Poon et al., 2016; Leonard et al., 2017).

This model provides one of the first rigorous investigations of transmission fitness for a virus that is directly transmitted between vertebrate hosts. However, due to its simplicity, this model also leaves out certain components of this biological system that could prove to be informative and helpful in interpreting the result. The main limitation in assuming viral exponential growth is that such formulation does not allow for the possibility that the success of one strain may depend on its frequency, thus preventing the possibility of mutant proportions in the recipient being lower/higher than in the donor for only some values of mixture proportion (i.e., data appearing on both sides of the *x* = *y* diagonal) (see Figure 2B). By having no implicit or explicit interaction between strains, only two scenarios of competitive exclusion are possible in this model: either strain 1 always wins (all points above the diagonal *x* = *y*) or strain 2 outcompetes strain 1 (all points below the diagonal), leaving no room for coexistence or bistability. This problem has persisted even in later adopted mechanistic formulations, e.g., Target cell -Infected cell-Virus (TIV) models, as they typically implemented the fitness variation between strains in a single parameter, e.g., through a difference in the production rate of infectious virus from infected cells (Butler et al., 2014), where again the principle of competitive exclusion applies.

### 2.2. A More Complex Alternative: The Lotka-Volterra Competition Model

One way to accommodate more complexity and generality into the system, is to develop an alternative approach for this type of competitive mixture data. We propose the next-order approximation away from the exponential model: a dynamic model based on the well-known Lotka-Volterra competition equations (Volterra, 1926), and interpret the transmission event as a snapshot from such competition dynamics. Let *n*_1_(*t*) and *n*_2_(*t*) be the number of virions of strain 1 and 2, respectively, at time *t* in the recipient host. They change with time according to the following equations:

(2) $$\begin{array}{l} \frac{dn_{1}}{dt}=r_{1}n_{1}-c_{11}n_{1}^{2}-c_{12}n_{1}n_{2} \end{array}$$

(3) $$\begin{array}{l} \frac{dn_{2}}{dt}=r_{2}n_{2}-c_{22}n_{2}^{2}-c_{21}n_{2}n_{1} \end{array}$$

At any given time, virions of strain *i* grow at a constant rate *r*_*i*_, compete with virions of the same strain with strength *c*_*ii*_ and compete with virions from the other strain with strength *c*_*ij*_, for *i* = 1, 2 and *j* = 2, 1. The strain-specific growth rate *r*_*i*_ could be seen as how quickly each strain reaches its carrying capacity, here represented by *K*_*i*_ = *r*_*i*_/*c*_*ii*_. A strain grows bounded by its carrying capacity due to limiting factors such as finite resources or space, according to a logistic growth in the absence of the other strain. Explicit viral infection kinetics is known to involve target cell limitation, which can play a key role in competition within and between strains. Different tissue tropism could be a mechanism for strain-specific target cell limitation. Intra-strain interactions could, on the other hand, be mediated via subtle antigenic cross-reactivities among strains, or susceptibilities of infected cells to coinfection by competitor virus. Here, for the sake of generality, we chose to only represent the net effect of such processes abstractly via the nonlinear competition terms. The values of the parameters are assumed to be constant in time. The transmission event is interpreted as an instantaneous inoculation from the donor at time *t* = 0, where the proportion of each strain is known/fixed from the experimental setup (*p*). In other words, by denoting the mutant as strain 1 and the WT as strain 2, we assume the proportion of the mutant strain in the donor as *n*_1_/(*n*_1_ + *n*_2_) at time *t* = 0, and the proportion of the mutant strain in the recipient host as *n*_1_/(*n*_1_ + *n*_2_) at time *t*, the observation point.

**Figure 3:**
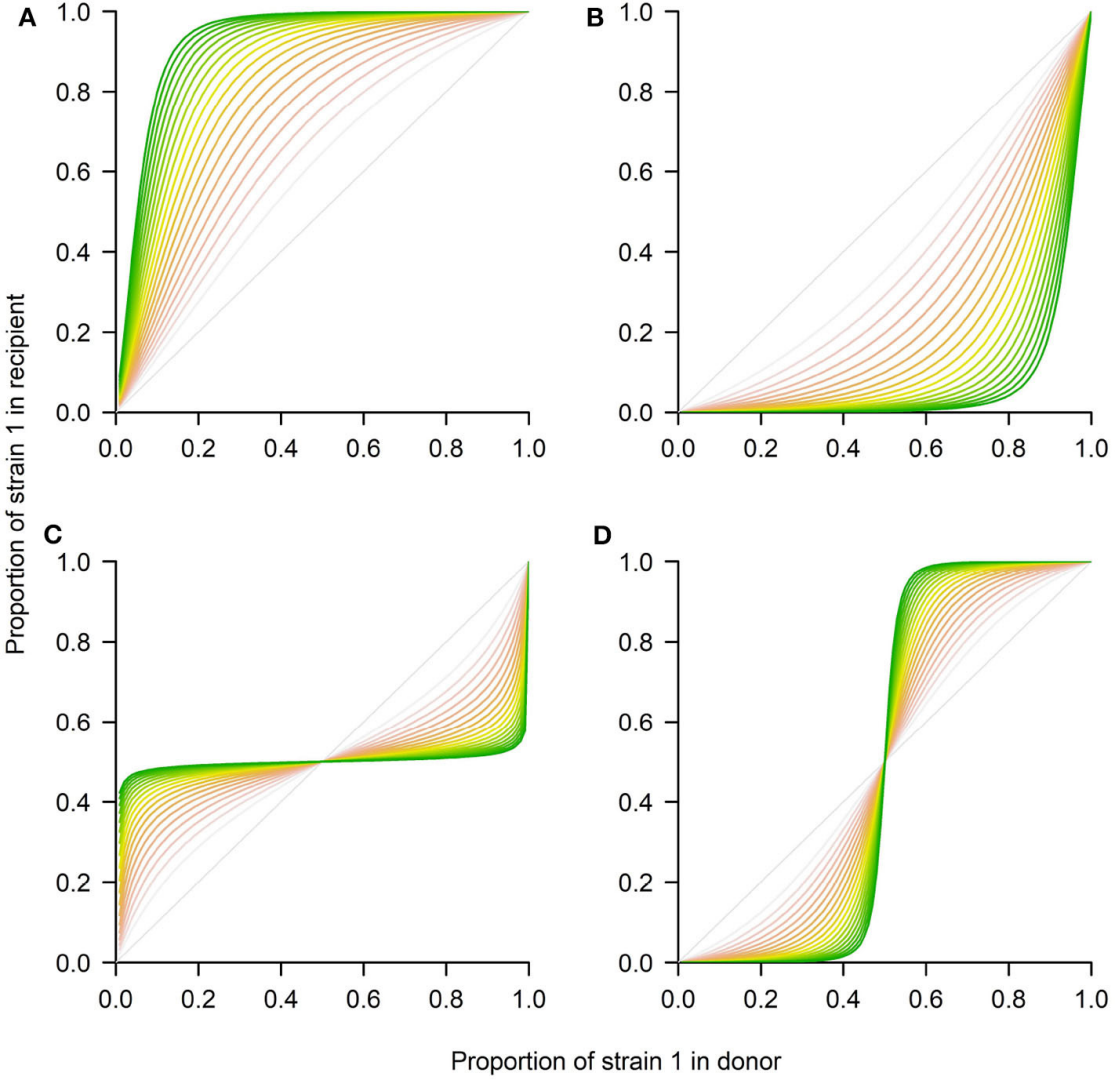
Four possible outcomes between 2 strains in transmission using the Lotka-Volterra model. The system of Equations 4–5 was numerically integrated using the *deSolve* package in R to represent the four ecological scenarios in terms of *p*_1_(τ) in the recipient. **(A)** Competitive exclusion where the mutant wins (strain 1). **(B)** Competitive exclusion where the WT wins (strain 2). **(C)** Coexistence between strains. **(D)** Bistability, where mutant or WT may win, depending on initial frequency. The different shading from red to green corresponds to later time-points over the donor-to-recipient dynamics (τ ∈ [0, 20]). As time increases, the system tends to approach more closely the equilibrium. Parameters values: **(A)** ρ = 1, *a*_12_ = 0.9, *a*_21_ = 1.3, β = 1. **(B)** ρ = 1, *a*_12_ = 1.3, *a*_21_ = 0.9, β = 1. **(C)** ρ = 1, *a*_12_ = 0.7, *a*_21_ = 0.7, β = 1. **(D)** ρ = 1, *a*_12_ = 1.3, *a*_21_ = 1.3, β = 1.

In order to reduce the number of parameters, a non-dimensionalization of the model is carried out. This implies re-writing the original parameters in Equations (2)–(3) as combinations of each other, thus reducing the total number of parameters from 6 to only 4. The model then becomes:

(4) $$\begin{array}{l} \frac{du_{1}}{d\tau }=u_{1}\left(1-u_{1}-a_{12}u_{2}\right) \end{array}$$

(5) $$\begin{array}{l} \frac{du_{2}}{d\tau }=\rho u_{2}\left(1-u_{2}-a_{21}u_{1}\right) \end{array}$$

where the new variables $u_{1}=\frac{n_{1}}{K_{1}}$ and $u_{2}=\frac{n_{2}}{K_{2}}$, denote relative densities of each strain with respect to their competitive exclusion carrying capacities *K*_1_ = *r*_1_/*c*_11_ and *K*_2_ = *r*_2_/*c*_22_. The new parameters ρ = *r*_2_/*r*_1_ and $a_{12}=c_{12}\frac{K_{2}}{r_{1}}$ and $a_{21}=c_{21}\frac{K_{1}}{r_{2}}$, capture the growth rate ratio between strains, and their relative competition indices. The ratio between the two carrying capacities is denoted by β = *K*_1_/*K*_2_. Notice that time in the rescaled model is also scaled to the new time-scale τ = *r*_1_*t*. The asymptotic analysis of this classical system is well-known, and only summarized here. In particular, this model allows for 4 scenarios (see Figure 3), depending on parameter values:

1. If *a*_12_ < 1 and *a*_21_ > 1, strain 1 outcompetes strain 2 (competitive exclusion of 2)
2. If *a*_12_ > 1 and *a*_21_ < 1, strain 2 outcompetes strain 1 (competitive exclusion of 1)
3. If *a*_12_ < 1 and *a*_21_ < 1, both strains coexist at a stable coexistence fraction (coexistence)
4. If *a*_12_ > 1 and *a*_21_ > 1, coexistence is unstable, and either strain 1 or 2 wins, depending on initial conditions (bistability).

There is an interesting prediction in relation to the coexistence threshold that this model makes for the last two scenarios. In particular, in the case of coexistence (Figure 3C), for any initial proportion below the *stable* coexistence fraction, the mutant will grow, and for any initial proportion above it, the mutant will decline, until it tends to the final equilibrium. In contrast, in the bistability case (Figure 3D) for any initial proportion below the *unstable* coexistence point, the mutant will decline toward 0, while for any initial proportion below such threshold, the mutant will grow toward 1.

Note that the rescaled variables *u*_1_ and *u*_2_ do not correspond to exact within-host proportions anymore. For the proportion of the mutant in the recipient at time τ we have *p*_1_(τ) = *u*_1_(τ)/(*u*_1_(τ) + *u*_2_(τ)/β), which is the model prediction from dynamics (Equations 4–5) in the recipient.

Assuming for the initial time point that *p*_1_(0) = *u*_1_(0), in the re-scaled model, this translates to an initial condition *u*_2_(0) = β[1 − *u*_1_(0)], where β corresponds to the ratio of the carrying capacities *K*_1_/*K*_2_. To apply this model to data, requires an assumption about the time of observation of mutant proportions in the recipient, which is typically experimentally-informed, and we assume a single snapshot exists. There are thus only 4 constituent parameters θ = (ρ, *a*_12_, *a*_21_, β), that can inform us about the behavior of this system in very distinctive way.

With this setup, we first carried out a simulation approach to validate the parameter estimation and their identifiability from a mixture dataset like the one modeled by McCaw et al. (2011), thus assuming only data on proportions are available, at a single time-point (we assumed τ = 1 without loss of generality), and no total counts of strain-specific viral loads. The error function to minimize between model and data is given by

(6) $$\begin{array}{l} MSE\left(\theta \right)=\frac{1}{M}\overset{M}{\underset{i=1}{\sum }}{\left(P_{i}^{model}\left(\theta \right)-P_{i}^{data}\right)}^{2}, \end{array}$$

where $P_{i}^{data}$ denote the *i* − *th* observation in the recipient for mixture proportion *i* in the donor (*i* = 1, ..*M* with *M* the number of data points), and $P_{i}^{model}\left(\theta \right)$ denote the model-prediction for that condition. Errors are assumed to be normally distributed. Using simulations with known parameters, we found that with such dataset, having sufficient mixture conditions around the 50–50% in the donor, the model and optimization algorithm could reliably estimate all 4 model parameters, and accurately classify the ecological competition scenario that applies between two strains (Figure S1). Except where stated, all data and numerical analyses were carried out in MATLAB and its optimization toolbox (MathWorks, 2011).

## 3. Results

### 3.1. Fitting the LV Model to Data

**Figure 4:**
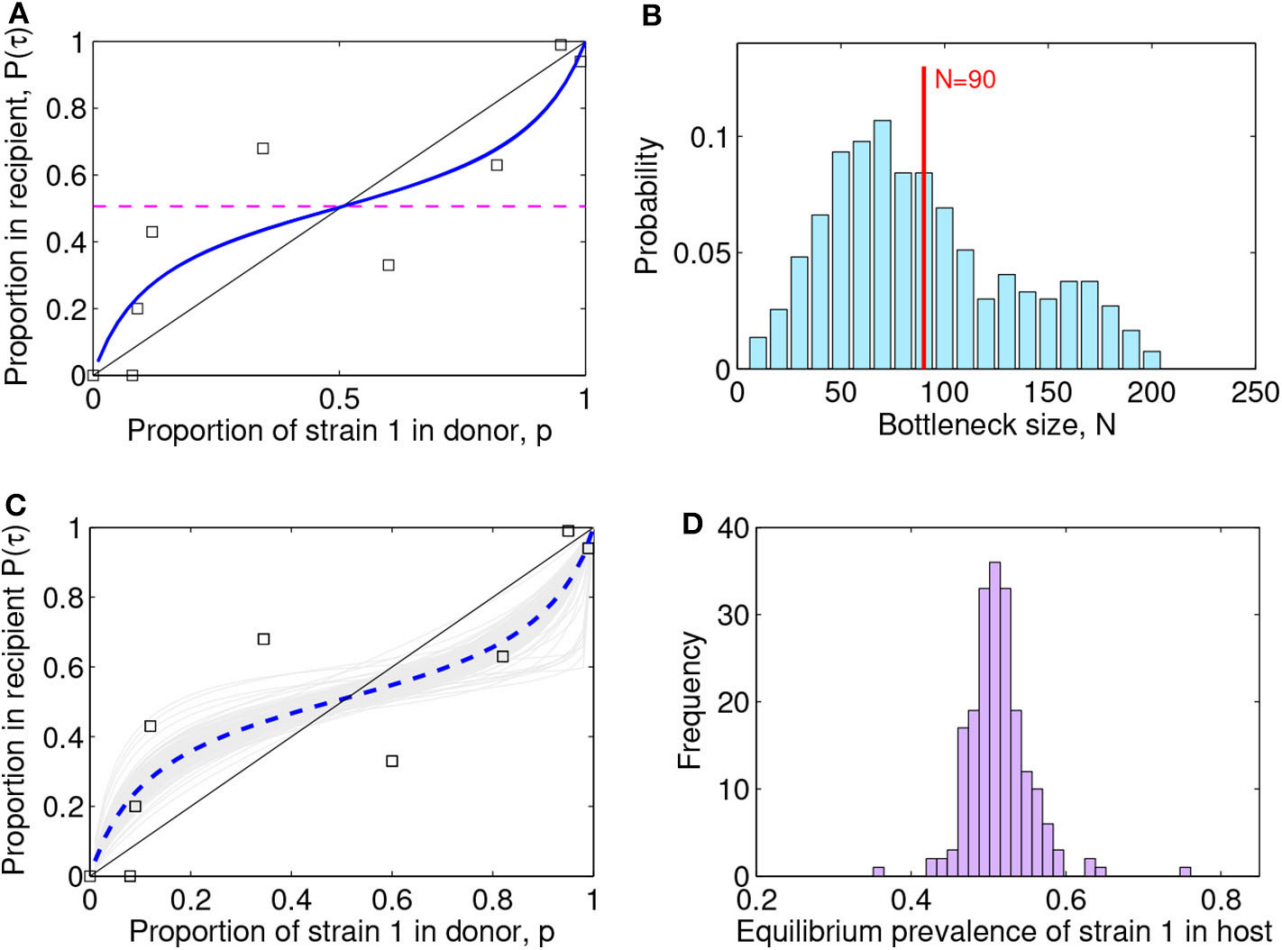
Lotka-Volterra model fit to the (McCaw et al., 2011) ferret transmission data. **(A)** Fit result with best parameter estimate [ρ, *a*_12_, *a*_21_, β] = [1.00, 0.25, 0.31, 0.94], MSE = 0.022. The squares indicate the data, the blue line the model fit, and the dashed purple line the predicted within-host coexistence equilibrium between the two strains. **(B)** Maximum-likelihood bottleneck size estimation *N* after filtering simulations and fits. The values of *N* ∈ [10, 200] were ranked based on: (i) the model quality of fit (mean-squared-error of new fit within 20% of the original MSE) and (ii) the capture of the data (proportion of original data points contained within the range of ten *N*-based stochastic simulations). We multiplied the two criteria for ranking *N*, and applied a moving average to smooth over *N*, and a standard normalization to transform the values into probabilities. The mean value estimated for the bottleneck is *N* = 90 (vertical red line) and the standard deviation is 45, thus suggesting the range *N* ∈ (45, 135) as the most compatible with these data and this model. The mode of the distribution is *N* = 70. **(C)** Visualization of 200 model fits (gray lines) to simulated data, varying the initial proportion transmitted from donor to recipient according to bottleneck sizes *N* sampled from the distribution in **(B)**. The dashed blue line indicates the mean over all the simulation fits. The squares indicate the data. **(D)** Model predictions for the within-host prevalence of the mutant strain in coexistence (Equation 8), integrating stochasticity and variation expected from *N* in **(C)**.

With the above setup and preliminary validation, the non-dimensionalized Lotka-Volterra model was fitted to the H274Y experimental data in McCaw et al. (2011) (Figure 4A). We denote the mutant influenza strain by *u*_1_ and the wild-type by *u*_2_, with *M* = 8 data points, namely mixture proportions tested experimentally. The time assumed for observations in the recipient was τ = 2. Considering that the time passed since strain measurement in the donor ferret and measurement in the recipient ferret was about 2 days in the experiments (Hurt et al., 2010), fixing rescaled time τ = 2 in the model corresponds to assuming a reference *r*_1_ = 1 for the mutant strain.

The best-fitting parameters (see first row of Table 1) yielded a mean-squared-error (MSE) of 0.022 (Figure 4A). Notice this mean-squared-error of the model fit to data is 40% lower than the best-fitting model parameters in the McCaw et al. (2011) exponential framework (MSE = 0.037), indicating a higher-quality fit of this model to the same data. The non-dimensional Lotka-Volterra model fit for these data is consistent with the scenario of coexistence within-host between the two strains of influenza, where although there is no fitness advantage of the WT in growth (ρ ≈ 1), the competition coefficients (*a*_12_ < 1 and *a*_21_ < 1) with *a*_12_ < *a*_21_ are compatible with mutual coexistence and a slight advantage of the mutant. This model is able to flexibly capture the pattern of some mutant proportions in the recipient being higher and some lower than in the donor, and predicts that over time the competition between strains should settle at

(7) $$\begin{array}{l} u_{1}^{*}=\frac{1-a_{12}}{1-a_{12}a_{21}},\;u_{2}^{*}=1-a_{21}u_{1}^{*} \end{array}$$

which leads to an expected equilibrium mutant proportion of

(8) $$\begin{array}{l} p_{1}^{*}=\frac{u_{1}^{*}}{u_{1}^{*}+u_{2}^{*}/\beta } \end{array}$$

which is ≈ 0.50 in our model (see horizontal dashed line in Figure 4A), at least in the early time-frame until immunity has not yet been activated, usually 2–5 days post-infection (Tamura and Kurata, 2004; Baccam et al., 2006; Smith et al., 2010). The model also infers that the ratio of within-host carrying capacities of the two strains is around 1 (β = *K*_1_/*K*_2_ = 0.93), thus suggesting that density-dependent regulation when each strain grows alone, acts quantitatively in similar way. In fact, these estimates capture the pattern of initial mutant growth advantage when rare, because it experiences less competition from the wild-type (points above the diagonal *x* = *y* for low mixture proportions, in Figure 2), and growth disadvantage when frequent (points below the diagonal for higher mixture proportions). Coexistence results from intra-strain competition being relatively weaker than within-strain competition. The net effect that the two strains coexist in nearly equal proportions within host, is an outcome that could also be seen as very little fitness difference between the two strains, in line with the interpretation of results by McCaw et al. (2011).

**Table 1:** Summary of parameter estimates from nonlinear least squares optimization, applying the model to the original data (Figure 4A), without considerations of bottleneck size, and fits to simulations with an explicit bottleneck size *N* (Figure 4C), all leading to similar mean-squared-error (MSE) and predictions for within-host prevalence between the two strains.

	**ρ**	***a*_12_**	***a*_21_**	**β**	***MSE***	Equilibrium $p_{1}^{*}$
Point fit to original data	1.0016	0.2494	0.3154	0.9365	0.0221	0.5066
Mean (fits to simulations with *N*)	1.1170	0.2852	0.3273	0.9930	0.0255	0.5085
95%CI (fits to simulations with *N*)	1.0000	0.0839	0.0652	0.8339	0.0181	0.4541
	2.7328	0.5923	0.5695	2.0000	0.0337	0.5723

Where the mutant proportion is denoted by $p_{1}^{*}$.

### 3.2. Estimating the Transmission Bottleneck

Next, we extended the model to include the possible stochastic effects of a transmission bottleneck. The estimation of the bottleneck size is inevitably model-dependent. To include explicitly the number of virions transmitted *N*, between donor and recipient host, we added a stochastic sampling step in the *x*− component of the data. We thus implemented the following change for initial conditions, depending on *N*, based on the binomial model:

(9) $$\begin{array}{l} u_{1}\left(0\right)=\frac{Bin\left(N,p\right)}{N}. \end{array}$$

Keeping the mutant proportions in the recipient fixed, we then refitted the model to these resampled data, simulating 10 realizations for a given *N* (Figure S2). We investigated how much variability this model element would introduce into the dynamics, when varying *N* discretely between 10 and 200 (see Figure S3). We analyzed the simulations and model fits for each *N* based on how well their range captured the original data, and also on their mean-squared-error (MSE).

For each *N*, we thus computed how many stochastic realizations yielded fits with MSE within 20% of the MSE of the original fit. This produced a ranking for *N* skewed toward high values. When considering the other criterion, based on how many of the original data points were contained within the range of stochastic realizations with a given *N*, we observed the opposite trend: smaller values of *N* were favored, because of the higher spread induced by stochasticity at low population size. Combination of the two criteria, was implemented as a multiplication between these two trends, which led us to obtain a single final ranking for *N*, analogous to a likelihood. We found an intermediate range for *N* as most appropriate to capture the data points, while preserving sufficient accuracy (Figure 4B). The mean value we estimated for the bottleneck is *N* = 90, with a standard deviation of 45, which indicates the range *N* ∈ (45, 135) as the most probable number of virions transmitted from donor to recipient, compatible with this dataset and this model.

Using this distribution for *N* in stochastic simulations, we were able to finally obtain an uncertainty interval for model predictions (Figure 4C) and confidence intervals for the model parameters (Table 1). In all cases, the predicted scenario by the model was one of coexistence within host between the two strains (mutant and wild-type in our case), with an expected prevalence of about 50% of the mutant strain (Figure 4D), independently of initial conditions. This reinforces our conclusion that these data strongly indicate equalizing net fitness between the two influenza strains of McCaw et al. (2011) upon transmission among ferrets and their early growth dynamics, despite the presence of some differences in their intrinsic competition phenotypes.

Overall, our proposed classical Lotka-Volterra competition model and *proof-of-concept* application to this competitive mixture dataset with influenza strains, highlight how variation in model sensitivity and ability to deal with frequency-dependence shapes data interpretation, as well as parameter estimates encapsulating fitness differences or transmission bottlenecks. This framework may enable stronger quantitative inferences to be drawn from other studies of virus dynamics *in vivo* in the future.

## 4. Discussion

Aggregation of microbial life-history details into net average parameters is important and necessary in all mathematical models of infection dynamics. As a consequence, any parametrization that leads to a specific model structure inevitably biases fitness assessment from data and phenotype-based comparative analyses between different strains. While the exponential growth model, motivated from population genetics, has found a wide use in relative fitness assessment between viral strains, recent studies are highlighting the need for more nuanced approaches, and calling for more mechanistic descriptions of pathogen dynamics within-host, and the multiple fitness dimensions driving growth and transmission (Holder et al., 2011; Pinilla et al., 2012; Petrie et al., 2015).

In this work, we follow the same spirit. In contrast to the more detailed models, we have advocated for the use of the well-known Lotka-Volterra equations to model competitive mixture dynamics and transmission of viral strains (Figure 1). The advantages of this framework are its simplicity (4 parameters), generality (4 ecological scenarios), and wide-applicability, despite being only a next-order extension to the classical exponential model. Such model can serve as an intermediate tool of optimal complexity between the binary exclusion model for two strains on one extreme (McCaw et al., 2011), and more detailed mechanistic explicit resource-based within-host approaches, similar to Petrie et al. (2015) on the other extreme, which can become particularly cumbersome and hard to parameterize especially when extended to transmission fitness estimation between strains. In fact, the Lotka-Volterra model, by virtue of its generality and simplicity, beyond its well-known role in multi-species ecology (May, 2001), has increasingly started to be applied to multi-type microbial competition scenarios (Stein et al., 2013; Shen et al., 2019; Tepekule et al., 2019) to bridge with data and offer robust and easily interpretable parametrization for experiments.

Our study has implications for the epidemiology of coinfection, mathematical modeling, and for understanding experimental results in competitive-mixture designs in general. By embedding in the model the possibility of frequency-dependent competition and hierarchies between strains, we match in scope the original experimental design of Hurt et al. (2010), which by definition, uses multiple ratios between two strains to initiate an infection, and anticipates qualitative and quantitative differences in transmission precisely based on such frequencies. Notice that a classical exponential model, in order to detect frequency-independent fitness differences between two strains could rely on a single (e.g., 50:50) mixture experiment, and its mathematical solution for *s*: $ŝ=\ln\left(\frac{\left(1-P\right)p}{P\left(1-p\right)}\right)$ where *P* and *p* represent proportions observed in the recipient and donor, respectively.

Thus, the Lotka-Volterra model expands the range of possible scenarios that can occur between strains in the absence of (or prior to) explicit immune control, mediated by their phenotypic differences, including coexistence and bistability. With mutual coexistence as a plausible outcome for the competition dynamics between two strains at the within-host level, the population-level coexistence and evolutionary patterns should become even easier to explain, taking into account their simultaneous co-transmission from host to host (Alizon, 2013). Interestingly, the other scenario of bistability within-host suggests that depending on initial frequencies and stochasticity upon contact, one strain or the other may win and be transmitted, creating more population heterogeneity.

These two alternative scenarios, beyond competitive exclusion at the single host level, suggest high within-host diversity and low between-host diversity in one case (within-host coexistence at a typical fraction p^*^), and low within-host diversity but high between-host diversity in the other (bistability within host yielding “0” and “1” host types at the population level). In a recent study of influenza B transmission fitness differences between strains, even though the classical exponential model was applied, the data contained a signature of possible bistability for MUT-Y273, (Figure 4C of Farrukee et al., 2018). Such signature may indeed be missed without an *a-priori* framework by which it can be detected. Correctly quantifying and disentangling these two scenarios of maintenance of pathogen diversity: coexistence vs. bistability, also based on their clear signatures in competitive mixture data as shown in Figure 3, may be of paramount importance when designing control strategies for different pathogens.

Until now coinfection models for respiratory viruses (Pinilla et al., 2012; Petrie et al., 2015; Pinky and Dobrovolny, 2016; Pinky et al., 2019) have not exposed clearly the four outcomes between two strains that we highlight here. Mechanistically, these four outcomes could be potentially mediated by competition for target cells and mutually altered susceptibilities to coinfection by pairs of strains (see Gjini et al., 2016 for an epidemiological example). Increasing evidence supports the notion that viruses do not propagate as independent virions among cells, but instead interact through subtle mechanisms and structures that can lead to social-like virus-virus interactions (Sanjuán, 2017). It remains an open avenue for the future to derive these four scenarios in an explicit Target cell-Infected cell-Virus (TIV) model, and investigate which parameter differences would be necessary and sufficient for their generation.

Furthermore, estimation of bottleneck size, another crucial quantity at the within-to between-host interface (Gutiérrez et al., 2012; Li and Handel, 2014), is very tightly linked to the assumptions of the underlying model. The more flexible a model is to capture intricate non-linearity in the data, the less room there is for patterns to be assigned to stochasticity, for example to low starting inoculum size. Here we have shown that assuming Lotka-Volterra dynamics, we can estimate a bottleneck size for number of virions transmitted between hosts most likely in the range of 45–135 virions, a number able to generate enough stochastic variation to capture the majority of the spread in the data around the deterministic prediction line, and more consistent with recent literature (Poon et al., 2016; Leonard et al., 2017).

One caveat in the parameter estimation with our model, is that time of observation enters explicitly in the equations, and does not cancel out as in Equation (1). Although we assumed τ = 2, motivated by the experimental setup, and took a reference of *r*_1_ = 1, if we change the assumption for time passed from measurement of strain proportions in the donor to measurement in the recipient, we see that longer time assumptions tend to increase slightly the growth rate ratio between strains ρ = *r*_2_/*r*_1_ in the model, and also the absolute value of competition coefficients, although their ratio, and in general the coexistence scenario, remain stable (see Table S1). This is not surprising, since we know that as time increases, the system is naturally pulled further away from the diagonal line tending closer to its equilibrium (whichever it is), even for very small fitness differences between strains. If we had prior knowledge on the reference net growth rate *r*_1_ of one strain for example, this confounding factor could be disentangled, because then the units of time would become explicit (τ = *r*_1_*t*).

A way forward to indirectly calibrate the timescale in our model may be to use estimates of influenza virus *R*_0_ from previous models applied to *in-vivo* settings (e.g., Petrie et al., 2013), and link those to the exponential growth rate within-host (*r*_0_, i.e., *r* in our model) using the analytical approximation (Nowak et al., 1997; Lee et al., 2009). *R*_0_ denotes the number of productively infected cells derived from each productively infected cell at the beginning of infection, and so far, estimates for influenza *R*_0_ vary between 3 and 75 in humans (Baccam et al., 2006; Smith et al., 2010), 300-10^3^ in ferrets (Petrie et al., 2013), and around 9 in mice (Smith et al., 2018). The associated estimates for the viral exponential rate of growth in host within the first 2 days post infection, suggest a rate of 5–18 per day in humans (Smith et al., 2010), and 11 per day in mice (Smith et al., 2018). As the exact calibration of *r* falls beyond our immediate scope here, in Table S1, varying τ ∈ [2, 6], we provide a crude sensitivity analysis for the time of observation effect, where model fits remain consistent with coexistence around 50%.

Other viral competition models have considered explicit target cell infection rate, viral production rates, and other detailed parameters (Perelson, 2002; Petrie et al., 2013, 2015; Butler et al., 2014), which complicate translation to the parameters of our formulation. However, alternative approaches (Smith et al., 2010), in contrast to such multi-parameter mechanistic formulations, have also recognized the utility of approximate analytic representations for different phases of viral kinetics within host (exponential growth, exponential decay), and used regression analysis to obtain the slope of viral growth and decay. Subsequently, these estimates have been related to intracellular infection parameters or the basic reproduction number. More recently, density-dependence has been suggested as an important element to be added to the classical exponential model, in support of a biphasic viral decay (Smith et al., 2018). Given this context, our addition of the Lotka-Volterra model, to the toolbox of simpler viral models, follows the same top-down aggregation spirit, and helps bring quantitative insight on key features of competition dynamics at the growth-transmission interface of mixed infections.

**Figure 5:**
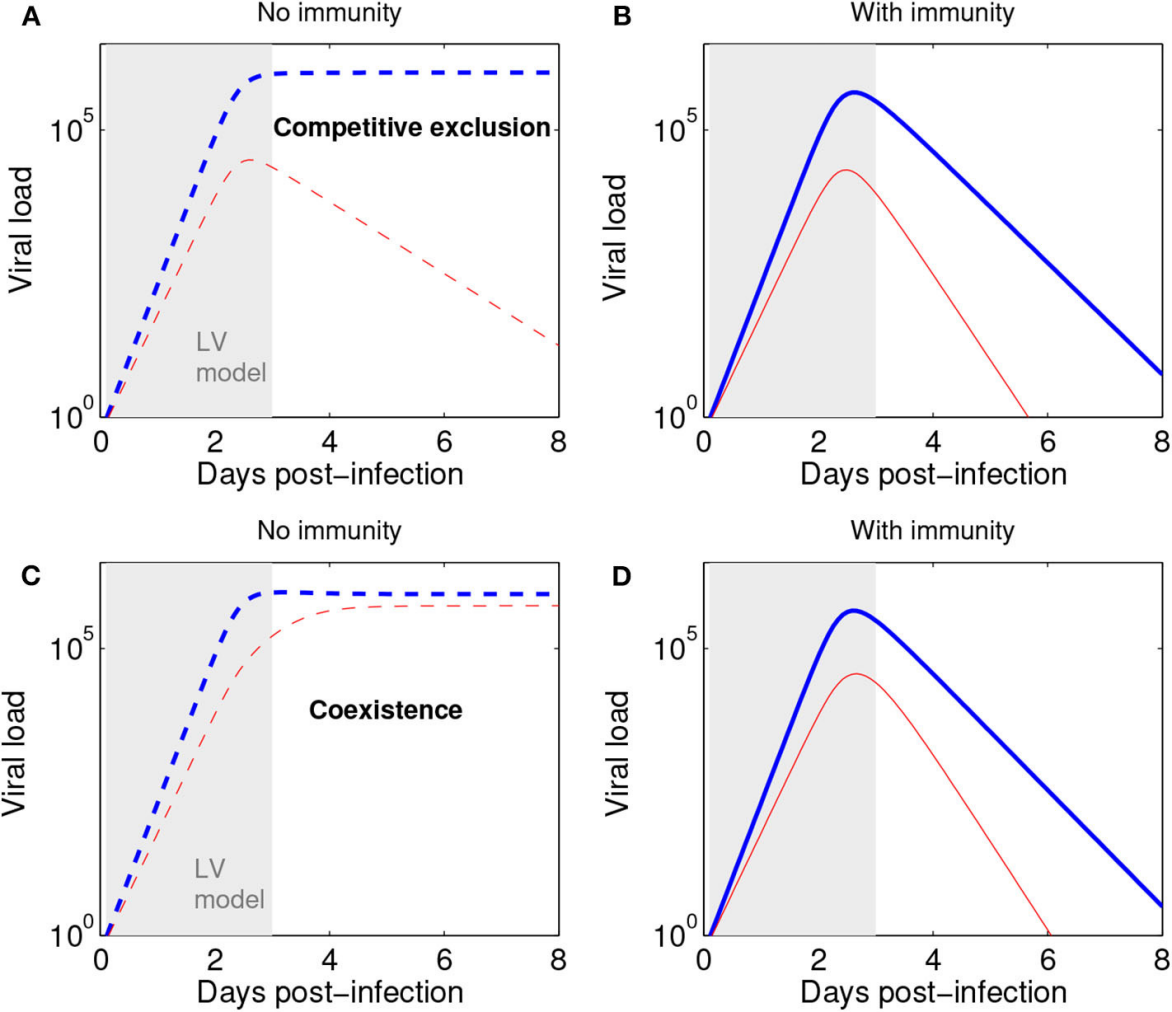
The Lotka-Volterra model can be augmented with action of the immune response to capture later infection stages. **(A)** Scenario of competitive exclusion (Equations 2–3 in this paper). Parameters, chosen to represent a realistic influenza infection kinetics (e.g., Farrukee et al., 2018; Smith et al., 2018): $r_{1}=6;r_{2}=0.8r_{1},c_{22}=r_{2}/10^{6},c_{11}=r_{1}/10^{6},a_{12}=0.9;a_{21}=1.3$ (blue: *n*_1_, red *n*_2_). *n*_1_(0) = *pN*_0_, with *p* = 0.5 (50:50 ratio for strains 1:2) and *N*_0_ = 1. **(B)** Scenario of competitive exclusion but with the addition of an immune response, besides Equations 2–3. A third equation for immune response growth kinetics can be: *dI*/*dt* = σ(*n*_1_ + *n*_2_) with *I*_0_ = 0, and σ = 0.01. The rest of parameters as in **(A)**, but adding a mass-action killing term :−*dIn*_*i*_ to each strain dynamics in Equations 2–3, where *d* = 10^−2.8^ denotes the rate of viral elimination by the immune response. Such increasing immune response as a function of viral load leads to an acute infection profile, and a total duration of about 8 days. Here viral load is represented in abstract units; in practice, it may be measured in units of TCID_50_ or rRT-PCR. **(C)** Scenario of coexistence (Equations 2–3 in this paper) Parameters: $r_{1}=6;r_{2}=0.8r_{1},c_{22}=r_{2}/10^{6},c_{11}=r_{1}/10^{6},a_{12}=0.2;a_{21}=0.5$ and initial conditions as in **(A)**. **(D)** Scenario of coexistence adding immune response (as in **B**). The initial dynamics within 3 days post infection (shaded region) are very well captured by the Lotka-Volterra model also in the presence of an immune response leading to ultimate infection clearance.

We remark that the Lotka-Volterra formulation for the initial phase of viral (or microbial) growth can be combined with subsequent action of the immune response to obtain more refined acute infection dynamics at later stages. As shown in Figure 5, with or without immunity (as assumed in our model) the initial phase of a mixed strain infection (within 3 days post-infection) is quite well-captured by our model (Equations 2–3), thus lending support to its more general suitability and applicability for later infection stages, as an alternative to the purely exponential growth model (Smith et al., 2010; McCaw et al., 2011). Recall that exponential growth is just a special case in the Lotka-Volterra model, when all competition coefficients *c*_*ij*_ are zero.

We based the estimation of the rescaled model parameters just on the availability of proportion data at one snapshot in time, like in McCaw et al. (2011), to highlight more the discriminatory power of the framework, even with minimal data. This precludes the full identification of the six parameters of the explicit model (Equations 2–3). When using both proportion data and total viral count data, over more time points, full parameter identifiability should be possible (see **Text S1**), with no need for rescaling and non-dimensionalization. Naturally, separating signal from noise remains a challenging problem in all areas of parameter estimation and model fitting to data, but when using a more complex model and when having confidence in the quality of the data, one can test for more complex biological signal and examine more refined hypotheses. Thus, although this work is a step forward in terms of proposing alternatives to modeling, it should also be taken as a call for higher-resolution data to probe more complex scenarios of fitness differences and outcomes between pathogen strains.

## Data Availability Statement

Publicly available datasets were analyzed in this study. This data can be found here: https://journals.plos.org/ploscompbiol/article?id=10.1371/journal.pcbi.1002026.

## Author Contributions

AD performed initial simulations, figure preparation, and contributed to the analysis of results. EG conceived and supervised the research and wrote the manuscript. Both authors contributed to the article and approved the submitted version.

## Conflict of Interest

The authors declare that the research was conducted in the absence of any commercial or financial relationships that could be construed as a potential conflict of interest.
