# Supplementary material for: Modeling Competitive Mixtures With the Lotka-Volterra Framework for More Complex Fitness Assessment Between Strains
Source: Front Microbiol. 2020 Sep 22;11:572487. doi: 10.3389/fmicb.2020.572487 (PMC7536265; doi:10.3389/fmicb.2020.572487)
Supplement: Supplementary file 1 [file Data_Sheet_1.pdf]

Supplementary Material

Modeling competitive mixtures with the Lotka-Volterra framework for more complex fitness assessment between strains, by A. D. Martins and E. Gjini

(Correspondence: erida.gjini@tecnico.ulisboa.pt)

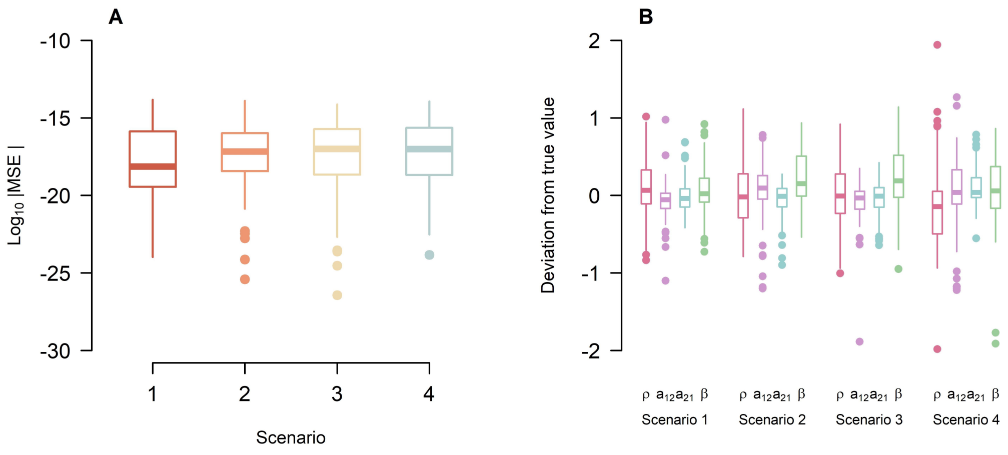

Figure S1. Validation of the parameter estimation procedure using synthetic datasets simulating different ecological scenarios. We varied model parameters to represent different ecological scenarios and assumed the time of observation was $\tau = 1$. We assumed initial mixture proportions of the same values as the data used in the paper. The scenarios simulated with Eqs. 4-5 in the paper, were: 1) competitive exclusion of mutant, 2) competitive exclusion of wild type, 3) coexistence, 4) bistability. We drew parameters randomly from the ranges: $\rho \in [0.01, 2], \beta \in [0.01, 2]$ and competition coefficients: 1) $a_{12} \in [0.01, 1], a_{21} \in [1, 2]$, 2) $a_{12} \in [1, 2], a_{21} \in [0.01, 1]$, 3) $a_{12} \in [0.01, 1], a_{21} \in [0.01, 1]$ and 4) $a_{12} \in [1, 2], a_{21} \in [1, 2]$. **(A)** Mean-squared-error (MSE) showing the quality of model fit across the four scenarios. The boxplots summarize 100 simulations for each scenario, and show the median, lower and upper quartiles, and the extreme lines reaching the minimum and maximum values excluding the outliers (shown as dots). **(B)** Parameter deviation from the true values across the four scenarios. In both the MSE and mean parameter deviation there are no statistically significant differences between the scenarios. Numerical investigation was performed in the R software environment (*deSolve* package and *optim* function (R Core Team, 2013)).

Table S1. Summary of parameter estimates from nonlinear least squares optimization applying the model to the original data, assuming a different time of observation in the recipient $p_1(\tau)$.

Time τ	ρ	a_{12}	a_{21}	β	MSE	Equilibrium p_1^*
$\tau = 2$	1.0016	0.2494	0.3154	0.9365	0.0221	0.5066
$\tau = 3$	1.0000	0.4758	0.5482	0.8793	0.0223	0.5050
$\tau = 4$	1.0000	0.5958	0.6632	0.8388	0.0225	0.5017
$\tau = 5$	1.0570	0.6693	0.7360	0.7958	0.0226	0.4992
$\tau = 6$	1.0251	0.7325	0.7782	0.8467	0.0226	0.5053

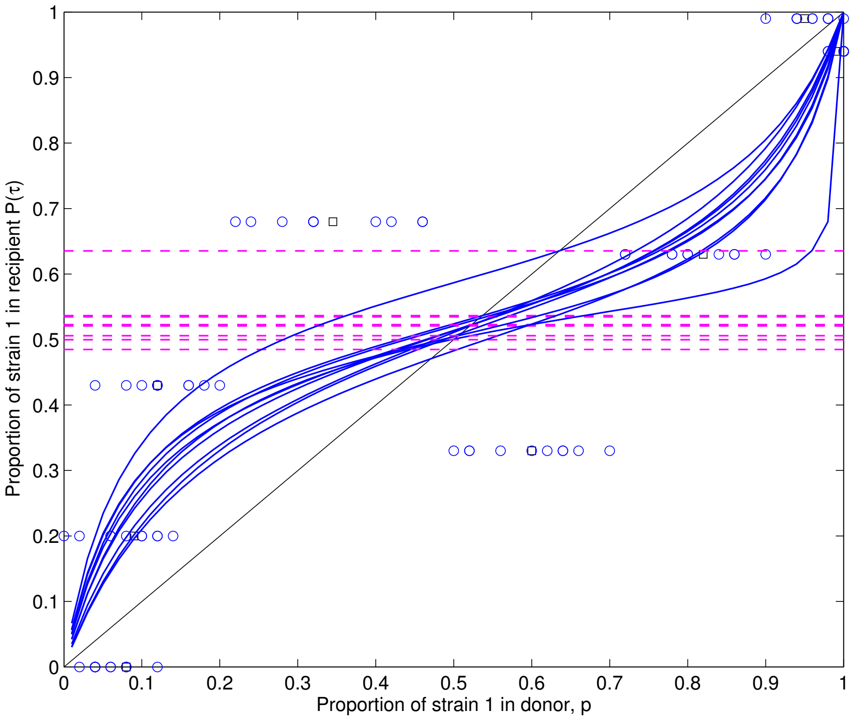

Figure S2. Illustration of bottleneck effect in the data resampling and model fits for $N = 50$ and 10 stochastic realizations. The black squares depict the original data, while the circles the re-sampled data accounting for bottleneck size N in the binomial distribution. The blue lines give model fits to each stochastic realization for this N. The dashed purple lines give the predicted equilibrium prevalence of strain 1 in the recipient for each realization.

Text S1: Lotka-Volterra Model written in terms of p and N

When substituting $N = n_1 + n_2$ and $p = n_1/N$, the model (Eqs. 2-3) becomes explicit in terms of proportion of strain 1, $p(t)$, and total population size $N(t)$:

$$\frac{dp}{dt} = p\Big[(r_1 - r_2)(1-p) - N(1+p)[c_{11}p - c_{12}(1-p)] + N(1-p)[c_{22}(1-p) + c_{21}p]\Big] \quad \text{(S1)}$$

$$\frac{dN}{dt} = N\Big[pr_1 + (1-p)r_2 - N[c_{11}p^2 + c_{22}(1-p)^2 + (c_{12} + c_{21}p(1-p))]\Big] \quad \text{(S2)}$$

It is clear that N affects p and p affects N in this system, thus it cannot be reduced. With data for total viral dynamics and fraction of strain 1 over several time points and initial conditions, the six parameters $r_1, r_2, c_{11}, c_{12}, c_{21}, c_{22}$ can be estimated using this version of the model. In case proportion data only are available, the non-dimensionalization trick in our Eqs. 4-5 can be used, or alternatively the replicator equation (Nowak and Sigmund, 2004) in 3-dimensions, which is topologically equivalent to the 2-dimensional Lotka-Volterra system (Bomze, 1995).

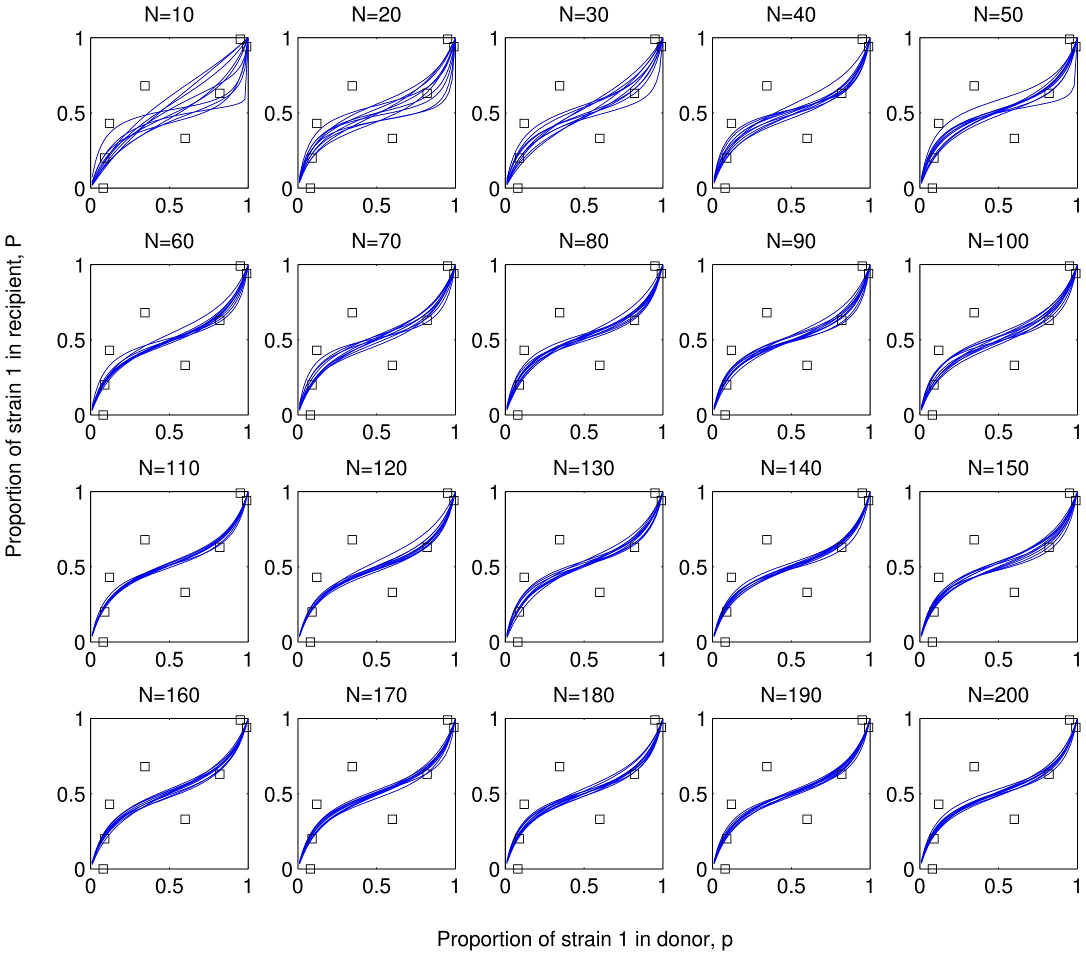

Figure S3. Model simulations and fits assuming different bottleneck sizes in the range 10 to 200. We vary the initial proportion that starts infection in the recipient according to a binomial sampling of the proportion of strain 1 in the donor. For each N we generate 10 stochastic realizations with different x-components of the data (fraction of strain 1 initiating an infection in the recipient), but same y-observations as in the (McCaw et al., 2011) study. Fitting the model to such data, we obtain the model fits (blue lines) with specific sets of parameter estimates for each N. As the bottleneck size increases, there is less variability around the deterministic model prediction and less data are captured by the spread in the simulations.

REFERENCES

Bomze, I. M. (1995). Lotka-volterra equation and replicator dynamics: new issues in classification. *Biological cybernetics* 72, 447–453

McCaw, J. M., Arinaminpathy, N., Hurt, A. C., McVernon, J., and McLean, A. R. (2011). A mathematical framework for estimating pathogen transmission fitness and inoculum size using data from a competitive mixtures animal model. *PLoS computational biology* 7, e1002026

Nowak, M. A. and Sigmund, K. (2004). Evolutionary dynamics of biological games. *science* 303, 793–799

R Core Team (2013). *R: A Language and Environment for Statistical Computing*. R Foundation for Statistical Computing, Vienna, Austria
